# The underuse of AI in the health sector: Opportunity costs, success stories, risks and recommendations

**DOI:** 10.1007/s12553-023-00806-7

**Published:** 2023-12-12

**Authors:** Ugo Pagallo, Shane O’Sullivan, Nathalie Nevejans, Andreas Holzinger, Michael Friebe, Fleur Jeanquartier, Claire Jean-Quartier, Arkadiusz Miernik

**Affiliations:** 1 Law School, University of Turin, Turin, Italy; 2 Department of Urology, Faculty of Medicine, University of Freiburg - Medical Centre, Freiburg im Breisgau, Germany; 3 Ethics and Procedures Center (CDEP), Faculty of Law of Douai, University of Artois, Arras, France; 4 Human-Centered AI Lab, Medical University of Graz, Graz, Austria; 5 University of Natural Resources and Life Sciences Vienna, Human-Centered AI Lab, Vienna, Austria; 6 Department of Measurements and Electronics, AGH University of Science and Technology, Krak’ow, Poland; 7 Faculty of Medicine, Otto-von-Guericke-University, Magdeburg, Germany; 8 Center for Innovation and Business Development, FOM University of Applied Sciences, Essen, Germany

**Keywords:** AI in Medicine, Underutilization of AI, AI Policy, Regulation of AI, Ethics of AI, AI Law

## Abstract

**Purpose:**

This contribution explores the underuse of artificial intelligence (AI) in the health sector, what this means for practice, and how much the underuse can cost. Attention is drawn to the relevance of an issue that the European Parliament has outlined as a "major threat" in 2020. At its heart is the risk that research and development on trusted AI systems for medicine and digital health will pile up in lab centers without generating further practical relevance. Our analysis highlights why researchers, practitioners and especially policymakers, should pay attention to this phenomenon.

**Methods:**

The paper examines the ways in which governments and public agencies are addressing the underuse of AI. As governments and international organizations often acknowledge the limitations of their own initiatives, the contribution explores the causes of the current issues and suggests ways to improve initiatives for digital health.

**Results:**

Recommendations address the development of standards, models of regulatory governance, assessment of the opportunity costs of underuse of technology, and the urgency of the problem.

**Conclusions:**

The exponential pace of AI advances and innovations makes the risks of underuse of AI increasingly threatening.

**Graphical Abstract:**

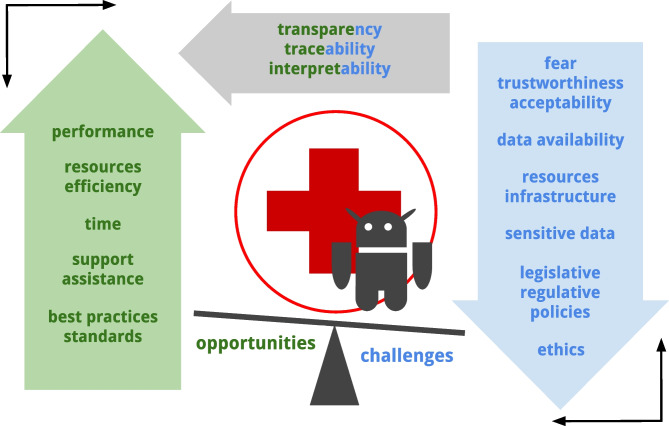

## Introduction

Over the past decades, scholars have dissected the manifold challenges of Artificial Intelligence (AI) in the health sector [1, 2]. The attention has been often drawn to possible misuses or overuses of technology that entail problems of privacy and security, fairness and equity, data quality and data aggregation, bias and transparency [3, 4]. These challenges clearly affect a pillar of the so-called international Bill of Rights, namely, the moral and legal right to health, as enshrined in Art. 25 of the Universal Declaration (UDHR) from 1948, and Art. 12 of the International Covenant on Economic, Social, and Cultural Rights (ICESCR). Scholars have accordingly stressed, “the need to adapt current evidence-based standards, to issues of privacy, oversight, accountability and public trust as well as national and international data governance and management” [5]. Whereas private companies, research institutions and national and international organizations with their public agencies have increasingly issued principles and guidelines for the use of AI in the health sector, lawmakers have been active too [6–8]. In April 2021, for example, the European Commission issued a proposal for a new Artificial Intelligence Act in EU law. Most of the medical devices, AI systems, and robotic applications under scrutiny in this paper should indeed be understood as ‘high-risk’ AI systems pursuant to the EU legislators [9].

In addition to threats and menaces of AI in the health sector that depend on misuses or overuses of the technology, the attention should be drawn to possible underuses of AI [10]. This is the subject of this paper. The claim is that the whole set of benefits and promises of AI can be missed or exploited far below its full potential in the health sector. For example, according to a press release of the European Parliament in September 2020, “underuse could derive from public and business’ mistrust in AI, poor infrastructure, lack of initiative, low investments, or, since AI’s machine learning is dependent on data, from fragmented digital markets [11]. Such drivers of technological underuse do not only regard easy replacements in current medical usages, but also new ones. Work in the field of Technology Use Theory provides the conceptual framework to dissect the “major threat” of AI underuse denounced by the European Parliament in its press release. Against this framework, we shall determine how much it costs to our societies not to use AI systems for health due to the wrong reasons.

The remainder is structured as follows:

First, Section 2 elaborates the theory of technological uses, followed by a discussion on the price for not using AI in health and a section detailing on current AI applications in health care. Section 5 addresses legal perspectives and other regulatory systems. Success stories are provided in Section 6 with insights into trends, followed by policy shortcomings and further to new recommendations for legal actors. The conclusion summarizes the limits but also lists recommendations for the future.

## The theory of technological uses

In its common sense, underuse corresponds to using less than what might be expected of a resource or technology. Non-use is considered a quasi-synonym of the term. In the field of technology use theory, the underuse of AI is directly linked to the notions of adoption, use and appropriation [12]. The notion of adoption consists in the acquisition of a technology, that of use refers to the concrete use of the technical object, while that of appropriation implies the technical and cognitive mastery of the tool [13]. The process of appropriation of a technique therefore corresponds to the transformation of a technology as it is envisaged by its designer into technology, as it is currently used [14]. Conversely, refusal and resistance to a technology are analyzed as non-use. The non-use of AI systems, factors of under-use, can be explained by external constraints by the specificities (missing trust, scepticism, fear, administrative burden with new innovations, and many more) linked to the clinical non-user (clinical staff, doctor or hospital).

In the case of external constraints, the reasons may be economic, budgetary or strategic. The cost of investment in hardware, software, updates, maintenance and the need to have staff trained in robots and AI systems can be seen as an economic and budgetary disadvantage justifying the refusal to acquire innovative yet effective tools. The health care facility may still hesitate in the face of internal financial and budgetary requirements and difficulties in funding innovation within the structure itself. For example, in France (and in other countries where EU purchasing rules apply), the complexity of the rules of the Public Procurement Code applicable to public hospitals is possibly to discouraging the acquisition of CAPEX (capital expenditure) intensive robots and AI systems. It is still possible that AI has not been identified as a strategic priority in the hospital. A 2019 survey of French university hospital centers shows that 24% of them believe that it is not really a priority for their own establishment[15]. This likely will have changed though in the recent months with the introduction of generative language models like ChatGPT. From a normative viewpoint, the underuse of AI is critical because advancements of technology can be slowed down, or even opposed for the wrong reasons. Professional reluctance, greed of both public and private data keepers, lack of standards and infrastructures, down to public disbelief in times of conspiracy theories are among the main drivers of technological underuse in the health sector [16].

Non-use of AI systems linked to the specificities of the non-user may find its source in the concerns of doctors who use AI systems as to the legal consequences in the event of damage caused to a patient [17]. Keep in mind that underuses of technology may also depend on legal regulations. They can either hinder the advancement of technology through strict liability rules for accident control, or through provisions that require over-frequent revision to tackle such progress [18]. In the EU, the proposal for a new Artificial Intelligence Act and the proposal for an AI Liability Directive of September 2022 create a comprehensive legal framework in which the European Commission considers appropriate to contribute to innovation and development in the field of AI, in particular by enabling professionals to anticipate risks.

The non-use related to specificities can also be largely explained by the lack of social acceptability of AI systems by doctors. For example, this is due to the complexity of using the AI system, the fear of the "black box" effect of Deep Learning, difficulties in evaluating and explaining the results provided by the AI system, concerns related to cybersecurity, fear of being replaced by the machine, resistance to change, fear of the dehumanization of the care relationship, and inadequacy of the AI system to the practices, etc. The 2019 survey carried out among French university hospital centers shows the ambiguity of the attitude of hospitals towards AI. While they are only a small majority to generally perceive the arrival of AI as very positive (57%), they overwhelmingly consider that AI is a very important subject for hospitals (81%). In all of these hypotheses related to technological acceptability, non-use then manifests itself either by an ab initio refusal to use an AI tool, or by abandoning or reducing use after having used the AI tool. However, even a dramatic drop can be reversible. For example, an adoption rate of an AI clinical decision support tool may drop dramatically due to an excessive system. By creating a tracking mechanism for monitoring trigger and adoption rates of a clinical decision support tool, one team found that adaptive modifications to the tool based on user feedback reduced trigger rates, thereby decreasing alert fatigue and increasing provider adoption of the tool [19]. Significantly, the acceptability of AI tools can improve when doctors see the tangible benefits. A study of the factors driving the adoption of a machine learning-based early warning system to detect sepsis proves that clinicians do not see the AI system as a substitute for their clinical judgment, but see themselves as a technology partner, even though they do not necessarily understand how this new tool works [20].

## The underuse of AI in the health sector

The ‘underuse of technology’ triggers that which economists call ‘opportunity costs’ [21], which in this context is the difference in productivity and quality gains of an AI supported health system and one that does not use or not use AI enough minus the cost for purchasing and maintaining the technology. The underuse entails lower standards in products and services, the redundancy or inefficiency of such products and services, down to the ‘shadow prices’ of the economy [22]. Work on national health services and their cost analysis estimated that the opportunity costs of ambulatory medical care in the U.S.A. are around 15%: “For every dollar spent in visit reimbursement, an additional 15 cents were spent in opportunity costs” [23]. In the U.K., the opportunity costs of the National Health Service may amount around 10 million pounds each year, whereas such figures could even underestimate the phenomenon [24]. Opportunity costs can be evaluated through thresholds for cost-effectiveness analysis [25], development of value frameworks for funding decisions [26, 27], etc. However, work on the opportunity costs that follow the underuse of AI systems for medicine and healthcare is in its infancy [9, 16]. The novelty of the issue, the difficulty of the task, the invisibility of the phenomenon, or the fact that scholars simply overlook the ‘major threat’ of AI underuse with its opportunity costs may explain the current state-of-the-art.

Accordingly, to appreciate how much it costs the underuse of AI in the health sector, the focus must be on the whole set of AI systems for diagnostics and prevention, precision medicine and medical research, clinical decision-making and mobile health, healthcare management and service delivery, down to AI applications for personalized health care. We would not discuss any underuse of AI for health if a panoply of such AI systems were not available out there.

### How to specifically address the laggards and sceptics?

It is very important to tackle AI implementations for example in a hospital as a group decision with stakeholders from nursing, administration, technical support, computer science, clinical staff, and also patient support. The first step in this direction concerns the digitization of data, starting from medical records up to the last possible complaint. Such digitization is not only indispensable for the use of AI systems in medicine and the health sector, but can effectively support functions and reductions of administrative burden as well as the reduction of costs that depend on traditional healthcare systems mainly hinging on paper records [28]. Several AI applications may help national health systems to dramatically decrease waiting times for specialist exams, unnecessary travels between home and hospital facilities, or people going to the emergency room of hospitals for unnecessary reasons. To specifically address the laggards and sceptics, it is worth mentioning that many of such AI systems regard administrative applications in healthcare. There are Robotic Process Automation (RPA) systems for medical records and revenue cycle management, clinical documentation, or claims processing [29]. Likewise, Natural Language Processing (NLP)-based systems can simplify such transactions as making appointments, or refilling prescriptions [30]. Other AI systems, such as Decision Support System (DSS) platforms embedding medical and AI algorithms can help decongest hospitals, by providing telemedicine services for homecare assistance [31]. The same holds true in the field of medical diagnostic investigation, in which AI systems can offer high quality services at low cost for preventive treatment services [32]. All in all, what all these examples show is that relatively easy installations can provide sizable and noticeable results, without posing any particular ethical dilemma or legal challenge. In addition to administrative applications, are there any further ‘low-hanging fruits’ for the use of AI systems in medicine and the health sector?

## A World of affordances

There is almost no field of medicine and health that is not affected by advancements of AI technologies. In the fields of diagnostics and prevention, for example, deep learning techniques have been employed to identify breast cancer [33], or sight-threatening retinal diseases [34], to predict severe sepsis [35], patients with COVID-19 [36], complications in intensive care units [37], or which populations are at risk for particular diseases [38]; in echocardiography, AI is used to diagnose coronary artery problems through people’s heartbeat [39], or to predict the incidence of heart failure in asymptomatic people [40]; in neurology, to predict and prevent cases of psychosis, either analyzing the patient’s linguistic and expressive behavior, or controlling her symptoms [41]. Further big data-driven applications of AI aim to warn specialists about high-risk conditions, such as cardiac arrest or infections [42], or populations that tend to require readmission to hospitals [43].

A whole set of its own regards AI systems for precision medicine and medical research. Machine learning techniques have been proven to be effective in predicting which treatment protocols are more likely to succeed based on the context in which the treatment must take place and the characteristics of the patients [44]. Likewise, AI systems have been employed for the detection of tumors and potential health risks detectable from medical record data and DNA analysis [38]. In China, a feasibility study tested the potential of AI systems in cancer research to define the most effective treatment for each patient [45]. In Africa, the SophIA project has used AI systems for the analysis of genomic data, to identify genetic mutations that can cause diseases and, hence, establish the best therapy [46]. In oncology, AI techniques for massive data mining have made it possible to explore the biomedical scientific literature in order to identify among the millions of studies’ implicit links that cannot be detected by a human [47]. Of course, algorithms do not only help in the diagnosis of the case but can (advise to) make decisions based on the identification of the problem. The collection of data from patient records and the corresponding clinical evaluation of the AI system allow some AI systems to make predictions in real time, providing all links and sources of information for appropriate recommendations. For example, some researchers at the University of Barcelona, Spain, have devised an AI predictor: on the basis of data taken directly from medical records in electronic format, the AI systems identify which hematological patients with neutropenic fever could have infections deriving from resistant bacilli to antibiotic treatments, the so-called MDR-GNB infections [48]. In France, a team of researchers has created a scoring algorithm capable of analyzing a CT scan of the lungs and five biological parameters to precisely calculate a severity score allowing the patient to be classified according to the probable evolution of their health, their risk of transfer in intensive care and the need for respiratory assistance [49]. The use of AI systems in medical diagnostic investigations and clinical decision-making often makes it possible to offer high quality services at low costs or in any case proportionate to the accuracy of the treatment services for prevention [32].

Further AI applications have been developed for healthcare management and service delivery [30], up to AI applications for personalized health care and mobile health. There are devices that, processing the information on the patient’s symptoms, can provide diagnoses [50]; suggest healthier lifestyles [51]; or control the use of medicines prescribed to subjects [52], as in cases of tuberculosis [53], mental illness [54], etc. These systems can thus provide personalized assessment about the health status of the user, consumer or patient, potentially reducing the demand for assistance from medical or healthcare personnel, relieving the pressure or tasks that often fall on the families, by offering forms of assistance that are effective, but at low cost, even in countries with few resources. Admittedly, the collection and processing of sensitive data by means of AI applications for mobile health trigger some of the issues mentioned above in the introduction with the protection of privacy and data protection, confidentiality and transparency of the data processing, consent and non-discrimination. In addition, the use of AI systems in the healthcare ecosystem often raises the difficulty of integrating such systems into the organization and workflows of the sector with its doctors, nurses, patients, etc. Remarkably, there is a hot debate on possible losses of jobs due to the replacement of personnel with various AI devices and systems [55]. Researchers may overcome hurdles of controlled, possibly sensitive health data for AI models by the use of synthetic data generation pipelines [56].

The digitalization of the health sector as well as the prediction, prevention, and personalization of medicine are in any event in the core of AI, e.g., machine learning techniques. Scholars have increasingly stressed the manifold ways in which AI systems can contribute to this paradigm shift in digital health. To previous samples of AI systems for diagnostics and prevention, precision medicine and medical research, clinical decision making and mobile health, healthcare management and service delivery, we may add work on the applicability of predictive diagnostics for the development of 3P-Medicine for clinical application [57]; AI supported patient self-care systems in chronic heart failure [58]; non-invasive diagnostic tools in coronary artery disease [59]; mass spectrometry-based technologies [60]; predictive diagnostics of dementia [61]; different stages of cellulite [62], and so forth.

Against this framework, we should question about how international organizations, national governments, and their public agencies aim to exploit the whole set of affordances and opportunities brought forth by AI for health, while protecting human rights and interests of all parties. In addition to declarations of principles and ethical guidelines, the focus of scholars has mostly been on acts and statutes against misuses and overuses of technology. For example, in EU law, the forthcoming Artificial Intelligence Act, the general data protection regulation, or GDPR, the machinery safety and cybersecurity regulations, the medical device regulation, etc. These normative acts represent a necessary, but insufficient element of the analysis. Such investigation should be complemented with the initiatives that have been taken by public agencies, governments, and organizations to tackle possible underuses of AI in the health sector with the soft tools of the law. The problem of how to implement the number of AI systems under scrutiny in this section can hardly be addressed on the basis of the top-down commands of hard law enforced through the threat of physical and/or pecuniary sanctions. The drivers of technological underuse, e.g., public distrust of the technology, cannot be simply tackled by legal order, or decree. What is then the current state of the legal art?

### Examples of uses of AI that have not been scaled up

Artificial intelligence (AI) has made significant strides in revolutionizing various facets of the healthcare industry. Nonetheless, despite the considerable advancements and widespread implementation of AI technologies in medicine, there remain several areas where its deployment is constrained or underutilized. An area that has yet to fully leverage AI capabilities is the development and integration of intelligent clinical decision support systems (CDSS). CDSS employs AI algorithms and machine learning techniques to analyze patient data, encompassing medical records, laboratory results, and imaging data. Although specific CDSS have been developed for certain medical conditions, their integration into routine clinical practice on a large scale is still to be accomplished. The scalability of these systems is impeded by challenges such as data interoperability, limited integration with electronic health records (EHRs), and concerns pertaining to liability and accountability [63]. The domain of drug discovery and development also represents an area where AI’s full potential has not yet been realized. Traditional drug discovery processes are costly and time-consuming, often entailing years of research and clinical trials. AI algorithms have demonstrated promise in expediting and optimizing various stages of this process. However, the broad application of AI in drug discovery and development encounters obstacles related to data availability, regulatory approval processes, and the imperative for interdisciplinary collaboration. By harnessing AI technologies, researchers can identify potential drug candidates, forecast their efficacy and safety profiles, and refine drug formulations [64]. Precision medicine aims to customize medical interventions based on individual patients’ distinctive genetic, environmental, and lifestyle factors. AI-based predictive analytics holds immense potential in actualizing the vision of precision medicine by discerning patterns and correlations within extensive datasets. However, the integration of AI into routine clinical practice for precision medicine remains limited. Challenges such as concerns regarding data privacy, the absence of standardized guidelines, and restricted access to advanced computational resources impede the scalability of AI-driven predictive analytics. Nevertheless, as increasingly comprehensive genomic and clinical datasets become available, coupled with advancements in AI algorithms, the feasibility of precision medicine applications, including disease risk prediction, treatment optimization, and identification of novel therapeutic targets, becomes increasingly viable [65]. The COVID-19 pandemic has underscored the significance of remote monitoring and telehealth technologies in delivering healthcare services. AI can play a pivotal role in remote monitoring by analyzing patient-generated data, such as wearable sensor data, physiological signals, and patient-reported outcomes, to identify anomalies and provide real-time alerts to healthcare providers. Despite the accelerated adoption of telehealth during the pandemic, the scalability of AI-driven remote monitoring systems is still limited due to challenges associated with data security, regulatory compliance, and reimbursement policies [66].

## The fight against AI underuse

The challenges of technological underuse - in particular, the underuse of AI systems for health - have recommended lawmakers to complement the hard tools of the law with the means of soft law, such as policies, guidelines, recommendations, and opinions of public agencies. Rather than a symptom of weakness, the soft tools of the law can be understood as an interface between the top-down instructions of the regulator, i.e., lawmakers and administrative agencies, and the interests of (some or all of) the stakeholders [67]. The US regulation of software as a medical device (‘SaMD’) illustrates this mix of hard and soft law with the powers of the Federal Drugs Administration [16]. It is up to the FDA to examine applications, develop policies, publish guidance, or ask for feedback. This mix of soft and hard law is at work also with the EU agency, EMA. The European Medical Agency shall evaluate applications for marketing authorisation, monitor the safety of medicines across their lifecycle, facilitate development and access to medicines, and provide information to healthcare professionals and patients. Much as occurs with further fields of legal regulation vis-à-vis the dynamics of technological innovation, e.g., AI drones in civil aviation law [68], the soft tools of the law appear particularly fruitful to enforce, strengthen, clarify, or stimulate the adoption of the top-down provisions of the regulator. In certain cases, e.g., development of standards, soft law provides for methods of coordination and cooperation to further define the content of the top-down provisions of the legislator. The engagement with some or all stakeholders with their feedback represents in many cases the only way in which legislators and public agencies can tackle the hurdles of technological innovation. Such stakeholders may include software engineers and computer scientists, patients and clinicians, academia and the industry, such as manufacturers and distributors, up to health technology assessments groups, the media and the public at large.

In the US, the FDA has set up patient outreach newsrooms and med watch, training modules and education programs, or networks with experts, e.g., regulatory associations. By interacting with health providers and educators, academia and the market, professionals and patients, the aim is to acquire reviews and contributions to reports, to learn about advancements of science and technology, and to inform stakeholders about policies, or to respond to requests related to the FDA authority [69]. Likewise, in EU law, the European Commission relaunched the ehealth stakeholder group initiative in July 2020, which refers to “all umbrella organisations/associations with a European outreach, representing the following sectors/groups: the health tech industry, patients, healthcare professionals and the research “community [70].” The intent is to "support the Commission in the development of actions for the digital transformation of health and care in the EU,” by providing advice and expertise, in particular, as regards the topics set out in the communication on enabling the digital transformation of health and care in the EU Digital Single Market, from April 2018. Such topics comprise some main drivers of AI underuse, such as health data interoperability and record exchange formats for digital health services through AI and “other cross cutting aspects linked to the digital transformation of health and care, such as financing and investment proposals and enabling “technologies [70].” In May 2022, the European Commission presented the proposal of the European Health Data Space, or EHDS, which covers a part, although important, of this challenge on the design and functioning of e-health record (EHR) systems through a mandatory self-certification scheme for such EHR systems.

Further initiatives such as the Medicines and Healthcare Products Regulatory Agency in UK, [71] the Health Sciences Authority of Singapore, [72] and the Department of Health of the Australian Government illustrate a similar approach against the underuse of AI. For example, the ‘Stakeholder Engagement Framework’ of the Australian Department of Health, provides for five principles of engagement that should help the law tackling cases of technological underuse through a clear understanding of the aims of the engagement, its inclusiveness, timeliness, transparency, and respectfulness, including expertise, perspectives, and needs of all stakeholders [73]. Five levels of engagement, that is, from simple information to consultation, involvement, collaboration, and delegation of legal powers to stakeholders, complement traditional top-down approaches of legislation [73]. Such forms of flexibility through different levels of engagement should allow the regulator to properly address cases of AI underuse that depend either on professional reluctance, or on public distrust and misapprehension, or on the difficulties to insert such AI systems into the organization and workflows of the health sector.

Yet, there is another formidable hurdle that legal flexibility and methods of cooperation and collaboration shall address in the fight against technological underuse, namely, public bureaucracy. The more legal systems rely on methods of coordination and cooperation to tackle the challenges of technological innovation, the more such methods of coordination and cooperation demand a new role of public agencies and authorities. Consider the engagement of stakeholders for tackling cases of technological underuse in medicine and health, the feedback that public agencies and authorities should have through such forms of engagement about the current state of the art, or the development of standards and metrics for the assessment of technologies that entails confrontation with developers and private companies. All these cases show that the role of authorities and public guardians is not only to enforce the top-down commands of lawmakers, but rather, to work together with all relevant stakeholders, such as private companies, non-governmental organizations, or the public at large, to find out solutions for an agile implementation of AI systems from labs to society. This approach often means, however, a change of mentality that, as many public agencies admit, can be as difficult as regulating the technology [16].

Traditional notions of legal compliance make this change of mentality even harder [74]. The old assumption, according to which either legal agents are compliant, or they are not, hardly fits the challenges of AI underuse and the corresponding policies of engagement, collaboration, and coordination under scrutiny in this section. Rather than 0s and 1s, between compliance and non-compliance, focus should be on more nuanced assessments that distinguish between ideal, sub-ideal and non-compliant statuses of legal agents [75]; or, between ‘good’, ‘ok’, or ‘bad’ compliance [76]; down to more fine-grained views that distinguish between average compliance, reasonably high compliance, very high compliance, and full compliance [77]. The binary alternative of compliance or non-compliance does not provide any useful information for the assessment and improvement of such institutional initiatives, as the soft law of the FDA, the ehealth stakeholder group initiative of the European Commission, or the ‘Stakeholder Engagement Framework’ of the Australian Department of Health.

In addition, compared to other regulatory systems in society, such as ethics and social norms, technology and the forces of the market, there is a paradox unique to the law. Legislators and initiatives of the public sector can be the cause of, or the solution for AI underuses in today’s human societies. On the one hand, many drivers of technological underuse do not depend on the law, but the law aims to govern them, on the other hand, the law can hinder technological innovation with its own provisions. Several examples of EU law in the regulation of e-money, drones, and now with the ‘high-risk uses’ of AI illustrate this risk, or impasse [18]. What is the state-of-the-art in health law?

## Success stories

This paper has stressed the pros and cons of AI in the health sector, its benefits and normative challenges. The threats not only regard misuses and overuses of AI, e.g., infringement of individual and group privacy, but also underuse of AI that depends on lack of infrastructures, connectivity standards, or the proverbial opacity and resistance of bureaucracy. How to strike a fair balance between affordances and constraints of AI is admittedly no easy task. Some success stories luckily illustrate how this balance is however feasible. For example, consider the developmental roadmap and validation of an AI service for health, i.e., CURATE.AI and foundational technology of IDentif.AI. The idea was to implement an AI system for “combination treatments where drugs administered together can interact with each other, which is often the case” [78]. The project moved from lab to ward, i.e., the National University of Singapore and its hospital, in a striking short amount of time in 2020. The project included engagement with the Medical Devices Branch of the Health Sciences Authority (HSA) in Singapore, that is, the regulator for risk classifications associated with such a device, since the very beginning of the project. For each trial and subsequent discussion for submission, rapid and informative responses and active engagement from HSA regulatory team members resulted in efficient turnaround times for trial initiation. Feedback from HSA ultimately resulted in a positive outcome for a refractory oncology patient [78]. A sustained track record of engagement with HSA made the whole process smooth, from lab to ward, playing a key role in helping a clear process flow to be developed for downstream guidance requests.

Remarkably, some of these success stories come also from low- or medium-income countries: AI systems that predict birth asphyxia in children by scrutinising the birth cry of a child via mobile phones in Nigeria [79]; AI apps that offer guidance and recommendations to nurses and paramedic personnel in India and sub-Saharan Africa [80]; or AI that detects water contamination [81]; or control dengue fever transmission [82]; or predicts Ebola outbreaks [83]. Such success stories show how making good through AI systems is feasible in the health sector. Human ingenuity provides for the means to implement in those settings technologies that are already used or have been developed in high-income countries, although within the specificities of each nation under scrutiny. The flexibility of the law that many western institutions have adopted through methods of coordination and cooperation - as illustrated above in the previous Section 5 - can thus address issues of technological underuse in developing countries, fleshing out which specific threats of AI underuse should be prioritized through initiatives that can be scaled up, or down, through the modularization of the projects [67]. This approach also fits high-income countries that have no experience of co-regulatory approaches, for example, Italy [16].

AI or respectively its workforce ML in cancer research offers novel perspectives such as ML clustering (with age or other groups) to find novel biomarkers [84]. In this domain, success stories include the use of ML enabling the largest Brain Tumor Study To-Date [85], as well as deep patient studies and derivates [86]. Some attempts indicate that AI-driven language systems can be used to correct misconceptions, to inform patients on cancer [87], and may be of further use in clinical scenarios [88]. A further perspective deals with opportunities regarding sustainability of XAI [89].

Success stories shall inspire models of governance that consider persisting crucial differences between countries and jurisdictions, between cultures and social norms. It is likely that striking differences among medical sectors, e.g., the opportunity costs of radiology vis-à-vis the opportunity costs of research in bacteria, should be expected in, say, Germany, France, or Spain. This conjecture rests, on the one hand, on traditional distinctions among medical sectors: we already stressed that, in medical diagnostic investigation and clinical decision-making, for example, AI systems provide for high quality services at low costs[32]. On the other hand, we should take into account clear differences among countries and their health services, for example, between the regional health services in Northern and Southern Italy. The result is that no single answer exists for the opportunity costs that follow ‘the’ underuse of AI in the health sector. Such opportunity costs will depend on the fields and types of AI systems under scrutiny, as well as on countries and jurisdictions taken into account. These constraints shall not make us overlook the inspirational source of all success stories and the general trend of technological regulation around the globe with a significant regulatory convergence between several health agencies. From Australia to the EU, Singapore, UK, or USA, public authorities have been setting up mechanisms of coordination, cooperation and co-regulation to cope with the challenges of technological innovation and risks of AI underuse. Some of these coordination mechanisms and methods of cooperation were at work with the success stories of this section. The next step of the analysis is to reflect on the overall achievement of such policies and initiatives against the underuse of AI for health.

## Policy shortcomings

We already illustrated the array of initiatives and policies of public agencies and national regulators that aim to prevent the risk of underusing AI systems for medicine and health. The premise of this paper was that the analysis on the underuse of AI for medicine and health does not revolve around whether such underuse exists, but rather, how much it costs to human societies. To corroborate the assumption, there is no need for personal experiences in hospitals and national health services around the world. Although generalizations must be avoided, according to the warnings of the previous section 6 on success stories, national and international institutions admit policy shortcomings in the fight against AI underuse. This paper already mentioned the 2020 press release of the European Parliament on the ‘threat of AI underuse.’ In the wording of the EU institution, “underuse of AI is considered as a major threat: missed opportunities for the EU could mean poor implementation of major programmes, such as the EU Green Deal, losing competitive advantage towards other parts of the world, economic stagnation and poorer possibilities for people.”

Similar threats of AI underuse have been underscored by further international organizations, such as the OECD, or ITU and WHO [9]. In the Tokyo 2019 AI Principles of the G20, the intergovernmental forum recommended a proactive approach of governments and public institutions to the risks of technological underuse. According to Art. 3.2(a) of the AI Principles, a proactive approach means that “governments should promote a policy environment that supports an agile transition from the research and development stage to the deployment and operation stage for trustworthy AI systems.”

Another very important but often hugely underestimated aspect is a technological effect: for certain tasks, algorithms can achieve performance beyond human levels, however, unfortunately, the most powerful AI methods suffer from the fact that, on the one hand, it is difficult to explain why a certain result was achieved. On the other hand, they lack robustness. In plain language, this means that the most powerful AI models are very sensitive to even small changes and perturbations. Thus, small perturbations in the input data can have a dramatic impact on the output and lead to completely different results - which of course can have fatal consequences in medicine and nursing - there is a lack of trustworthiness. This is precisely the reason that has led to an increased demand for trustworthy AI [90]. The lack of trustworthiness in this kind of technology strengthens the reasons why the affordances of AI systems are often missed due to wrong reasons, such as popular beliefs and mistrust on ‘black boxes’ technologies that encourage professional reluctance, bureaucratic resistance, or simply crazy conspiracy theories [91].

In the sensitive domain of medicine, traceability, transparency and interpretability are required. Some legal systems have already come a long way, with explainability now even mandatory due to legal requirements - for example, in the European Union [92]. Arguably, AI systems shall be made more robust and combined with explainability. Combining statistical machine learning with knowledge representations can help make AI models to be more robust. Certain tasks benefit from the inclusion of humans in the loop. Although not necessarily, but they often bring experience, domain knowledge, and conceptual understanding to the AI pipeline [92]. This may entail a virtuous circle. While including humans in the loop eases the liability burden of the legal system, the ‘why’ in many application areas is often more important than a pure classification result. Explainability and robustness can thus promote reliability and trust, and guarantee that humans remain in control when using AI systems for prediction, prevention, and personalization in medicine. The overall aim should be to complement human intelligence with artificial intelligence, i.e., the mission of the enormously growing field of Trustworthy AI [93].

The ‘agile transition’ from labs to society faces, however, formidable obstacles. The lower standards in products and services that follow as a result with the redundancy or inefficiency of some of such services have a cost. Consider the hours spent in unnecessary waiting times in hospitals or health facilities with the often-superfluous transport costs for patients, families and assistance staff, etc. We already noted above in the introduction that the evaluation of these costs is no easy task due to traditional hurdles of econometrics as well as the novelty of the phenomenon. Lack of standards and metrics does not only make it difficult to assess the opportunity costs of AI, but also the costs of its overuses or misuses. The footprint of AI, for example, is controversial as regards energy costs, carbon emissions [94], and the metrics of AI systems are optimized for, e.g., efficiency through model training [95]. In the health sector, such indexes, as the health-related quality of life (HRQOL), or the quality-adjusted life-year (QALF), enjoy a certain consensus, still, it remains unclear about the amount of the opportunity costs for the underuse of AI, also but not only, for medicine and digital health [96].

As of this writing, it is noteworthy that such international organizations and institutions, as the OCSE, the G20, or the European Parliament have not released any assessment on the opportunity costs that follow the underuses of AI under scrutiny in their own documents. Work on the AI underuse in the health sector suggests that in some countries, e.g., Italy, which invests around 9% of its gross domestic product (GDP) in the public health sector, the opportunity costs of technological underuse may amount to 1% up to 2% of the Italian GDP [16]. The figure includes the optimization of services, quality of standards, and the ‘shadow prices’ of people’s useless waiting lists and movements, traffic congestion, pollution, etc. The estimate also includes the costs for the modernization of public agencies and continuing education and formation of the personnel, as well as such trends as the increase in life expectancy, the spread of chronic or endemic diseases, and the greater familiarity that the new generations have with technological devices. AI systems look particularly promising to tackle these (social, legal, demographic, technological, etc.) trends with the full array of applications for medicine and health examined throughout this paper. The empirical assessment of the opportunity costs of AI is critical to determine public policies. We already noted that, especially dealing with medium- or low-income countries, or high-income countries with few or no experience of co-regulatory models, such as Italy, the first step is to specify what threats of AI underuse should be prioritized through initiatives that can be scaled up through the modularization of the projects.

Waiting for further work on the opportunity costs of AI in the health sector, we should not overlook, however, the ways in which current initiatives and public policies against the underuse of AI can be ameliorated, thus diminishing the costs of such underuse. This is another territory of work and research on the underuse of AI that legal theory and its corresponding models of legal governance have scarcely explored. The law can be a cause of, or a solution for technological underuses. Scholars have extensively debated new forms of legal governance and regulation for the challenges of AI and other emerging technologies, but rarely on how underuses of AI should be tackled through the means of binary governance [97], linked democracy [98], legal experimentation by derogation and by open access [99], co-regulation [100], algorithmic regulation [101], and more. The next Section 8 intends to fill this gap in discussions about the normative challenges of AI and its governance, proposing some recommendations to improve current policies and initiatives against the underuse of AI in the health sector. Empirical research on the underuse of technology and the quantification of its costs should be complemented with policy recommendations as to how to reduce such opportunity costs.

## New recommendations for an old paradox

All the recommendations in this section regard the specificities of soft law at work in complex digital ecosystems. The use of coordination mechanisms and methods of collaboration by governments and public agencies, as examined above in this paper, seems in fact indispensable in the fight against AI underuses. Drivers of technological underuse, such as public distrust and business diffidence, can hardly be addressed only on the basis of the top-down instructions of the regulator. The response of most public health agencies to the threat of AI underuse has thus intended complementing the binding rules of the law with mechanisms of coordination, methods of cooperation, etc. It is notable that, in EU law, specific norms are devoted to the functioning of such coordination mechanisms and the corresponding flexibility of the legal system. For example, in the data protection regulation, the GDPR has aimed to attain such aims of coordination with Articles 5, 60, 61, 75(4) and 97(2)(b); in the Chips Act from 2022, specific coordination mechanisms are set up with Art. 1(1)(c), Art. 14(4), and Art. 23(4), in accordance with the objectives of the Act (i.e., 1.4.2.3, at 63); etc.

This mix of soft law and hard law in the governance of complex digital ecosystems determines the extent to which the law may augment or decrease cases of technological underuse. Consider the success stories of this paper. What they have in common is not only the digital dimension of what ended well in all those stories, but rather, how the different actors of such stories exploited and benefited from the opportunities opened up by AI systems interacting on the internet although in an intricate social context. The constraints and affordances of the digital dimension of data-driven technologies, such as the use of AI systems, affect all stakeholders, from patients and clinicians to software engineers and computer scientists, manufacturers and distributors, health technology assessments groups and the public at large. Such impact of technology on the health sector also affects the way in which lawmakers and public agencies have conceived the governance of this sector. As declared repeatedly by the FDA in its 2020s reports, the impact of AI and further emerging technologies makes it necessary modernizing the organization of such agencies, as the FDA, fragmented in watertight scientific review processes [16]. Initiatives of engagement and coordination have similarly had to be reengineered. As shown by the focus groups set up by both ITU and WHO in their 2020-2022 AI for Health (AI4H) project, [102] traditional research on bacteria and dental issues, diabetes and endoscopies, radiology and malaria, has been complemented with the new data-driven scenarios of today’s biomedicine and AI that span across all traditional fields of medicine and health: analysis on software life cycle and data requirements, best practices and evaluation considerations, scale up and adoption of AI technologies, down to the assessment of AI applications and platforms.

The paper stressed that this digital component of the underuses of AI for medicine and health not only impacts the traditional organization of public agencies, but also the way in which the law should address the challenges of technology. The law should be ‘flexible’ especially when dealing with cases of technological underuse. The analysis has so far cast light on three different ways of legal flexibility, given by the means of soft law, different levels of engagement with stakeholders and methods of cooperation, and legislations that shall not hinder technological innovation nor require over-frequent revision to tackle the progress of technology. What all the variables of the analysis illustrate is, so to speak, the strength of soft law. Although not enforced by the threat of physical and pecuniary sanctions, as occurs with the tools of hard law, soft law can be effective. A certain degree of compliance by the legal actors involved in efforts of cooperation, coordination, etc., thus determines the success of a certain policy, e.g., the current fight against the AI underuse. The binary alternative of compliance or non-compliance, which makes of course sense when dealing with the top-down rules of hard law, does not provide, however, any useful information for the assessment and improvement of current institutional initiatives against the underuse of AI for health. A subtler, ‘more flexible’ approach to legal compliance in cases of AI underuse, also but not only in the health sector, shall provide more nuanced assessments than the traditional stance, according to which either the legal agent is compliant, or not.

Rather than 0s and 1s, subtler forms of evaluating the status of adherence to the regulatory provisions of the law are indispensable when using the soft tools of the law, or complementing the top-down commands of lawmakers with more flexible ways of legal coregulation. Such assessment regards also but not only the fight against the AI underuse for medicine and health. Consider the ‘popularity’ of the registration mechanisms for data altruism that have been set up with the Data Governance Act in EU law, as well as the ‘soundness’ of technological solutions for the principle of data protection by design, and by default, in several jurisdictions that endorse the principle [103]. The binary alternative of compliance or non-compliance does not help us improving current institutional initiatives that hinge on methods of coordination and cooperation since the alternative, or 0, or 1, does not clarify the nuances of such engagement. The principle of legal certainty that fits the hard tools of the law hardly adapts to the evaluation of the tools of soft law. This is particularly true when assessing the current fight against AI underuses for medicine and health.

The development of new legal standards, e.g., the assessment of legal compliance in cases of AI underuse, goes of course hand-in-hand with the development of new technical standards in medicine and health [104]. We noted that many fields lack standards and metrics not only for the assessment of the opportunity costs of AI, but also for the costs of its overuse or misuse. This lack of standards may trigger a vicious circle because the lack of standards is another crucial driver of technological underuse. Therefore, the development of new legal standards and technical standards is not only necessary but should be conceived of as the two sides of the same coin. On the one hand, such standards should provide thresholds for the assessment of the opportunity costs that follow the underuse of AI, and whether and to what extent current policies against the underuse of AI can be deemed as successful; on the other hand, new legal and technical standards for the use of AI systems can dramatically increase the protection of human rights in the health sector. This paper has mentioned manifold AI systems for predictive diagnosis, targeted prevention, or the personalization of medical services, as sound examples of how such uses of AI can enhance “a high level of human health protection” and the “right of access to preventive health care and the right to benefit from medical treatment,” according to the wording of Art. 35 of the European Union’s Charter of Fundamental Rights (CFRs). The aim should not only be to protect the right to health against misuses and overuses of technology, but moreover, to strengthen today’s standards of protection through the increasing use of trustworthy AI systems for health.

## Conclusions

This paper has mostly explored terra incognita, examining the underuse of AI for health, what it means and how much it may cost. Attention was drawn to the relevance of an issue that the European Parliament has presented as a ‘major threat’ in 2020. Scholars have increasingly debated the crucial role that AI innovation plays in the health sector, so that, drawing on this previous research, the focus has been on the risk that research and work in trustworthy AI systems for medicine and digital health may pile up in lab centers with no further impact. The analysis has dwelt on why scholars should be attentive to this phenomenon, inspecting the ways in which governments and public agencies have intended to tackle it. Since such governments and public agencies often admit the limits of their initiatives, this paper has examined the causes of this impasse, proposing some ways to ameliorate such initiatives. The exponential rate of AI advancements and the fact that prediction, prevention, and personalization are in the core of AI make risks of AI underuse increasingly threatening in this field [105–109].

Limits and drawbacks of today’s fight against the underuse of AI - also but not only for health - do not simply depend on the legal means employed by national governments and their public agencies, i.e., the soft tools of the law and methods of cooperation, rules of coordination, etc. Rather, the risk that an increasing set of AI systems for medicine and digital health may pile up in (the warehouse of) start-ups and research centers has to do with the lack of such coordination mechanisms and cooperation in several countries and jurisdictions. Even at their possible light, we should admit however that current policies and initiatives against the underuse of AI require time. Consider the modernization of such organizations, such as the FDA, or the WHO, or matters of public and business trust, or distrust, that are time demanding par excellence. Moreover, the development of new legal and technical standards for AI in medicine and health should not be expected to occur overnight. The assessment of misuses or underuses of AI systems, technical standards for connectivity and data processing, further standards of EU law for the class of ‘high-risk’ AI systems employed in medicine and for human healthcare, etc., should rather be understood as a part of the work that has to be done in the future.

This medium-term perspective does not preclude short-term recommendations on the ways in which current mechanisms of cooperation, soft law, engagement, and cooperation for the fight against the underuse of AI can be improved. These recommendations regard models of legal governance, standards, evaluation of opportunity costs, and the overall relevance of the problem, i.e., the ‘major threat’ of AI underuse for health.

The first recommendation has to do with a lesson learnt from medium- and low-income countries and their success stories. The first step is to clarify the specific threats of AI underuse that should be prioritized in a certain country. The initiatives should be scalable through the modularization of the projects. The model can be extended to high-income countries with no experience of co-regulatory approaches. The modularity and scalability of the approach should help the law tackling the mid-term issue of the modernization of public agencies and organizations.

The second recommendation regards the development of new legal standards. They shall include the finetuning of different degrees of legal compliance. The more public agencies and governments (properly) insist on the soft law tools of cooperation and engagement, the less a traditional binary approach is fruitful. We need future work on how to evaluate the success, or unsuccess of their policies, through different degrees of legal compliance, between 0 (compliance) and 1 (non-compliance).

The third recommendation brings us back to the evaluation of the opportunity costs that follow the underuse of AI systems in the health sector. We need future work on how much it costs not to use them, according to different classes and services of AI systems, in different jurisdictions, e.g., common law and civil law, and in different cultures and traditions, e.g., the civil law of France or Italy. Empirical research is critical to shed light on these differences and set up corresponding policies.

The fourth recommendation regards the urgency of the problem. It depends on the exponential growth and advancements of AI. By considering the flourishment of AI systems for health, the alternative to the use of trustworthy AI systems is not the simple protection of the status quo. Due to advancements of AI techniques and the speed of AI innovation, the alternative is abating the level of protection of today’s rights, exponentially. We should take the underuse of AI and its opportunity costs seriously. The underuse of AI can be grasped as a waste of time, money, resources, and quality of life. This is a human tragedy. No country can afford it.

## Data Availability

Not applicable.

## The following abbreviations are used in this manuscript:

AI: Artificial Intelligence
CAPEX: Capital Expenditure
CFRs: Charter of Fundamental Rights
CDSS: Clinical Decision Support Systems
DSS: Decision Support System
XAI: Explainable Artificial Intelligence
ICESCR: International Covenant on Economic, Social, and Cultural Rights
ML: Machine Learning
NLP: Natural Language Processing
RPA: Robotic Process Automation
UDHR: Universal Declaration
